# N-Containing Porous Carbon-Based MnO Composites as Anode with High Capacity and Stability for Lithium-Ion Batteries

**DOI:** 10.3390/molecules29122939

**Published:** 2024-06-20

**Authors:** Yi Cheng, Shiyue Li, Wenbin Luo, Kuo Li, Xiaofei Yang

**Affiliations:** 1 Hangzhou Institute of Advanced Studies, Zhejiang Normal University, 1108 Gengwen Road, Hangzhou 311231, China; luowenbin815@163.com (W.L.); 15904466605@163.com (K.L.); 2 School of Chemical & Environmental Engineering, China University of Mining & Technology (Beijing), Beijing 100083, China; shiyuel1998@163.com; 3 School of Light Industry and Chemical Engineering, Dalian Polytechnic University, Dalian 116034, China; 4 Division of Energy Storage, Dalian Institute of Chemical Physics, Chinese Academy of Sciences, Zhongshan Road 457, Dalian 116023, China

**Keywords:** Li-ion battery, biomass, MnO composites, self-template, porous carbon

## Abstract

MnO has attracted much attention as the anode for Li-ion batteries (LIBs) owing to its high specific capacity. However, the low conductivity limited its large application. An effective solution to solve this problem is carbon coating. Biomass carbon materials have aroused much interest for being low-cost and rich in functional groups and hetero atoms. This work designs porous N-containing MnO composites based on the chemical-activated tremella using a self-templated method. The tremella, after activation, could offer more active sites for carbon to coordinate with the Mn ions. And the as-prepared composites could also inherit the special porous nanostructures of the tremella, which is beneficial for Li^+^ transfer. Moreover, the pyrrolic/pyridinic N from the tremella can further improve the conductivity and the electrolyte wettability of the composites. Finally, the composites show a high reversible specific capacity of 1000 mAh g^−1^ with 98% capacity retention after 200 cycles at 100 mA g^−1^. They also displayed excellent long-cycle performance with 99% capacity retention (relative to the capacity second cycle) after long 1000 cycles under high current density, which is higher than in most reported transition metal oxide anodes. Above all, this study put forward an efficient and convenient strategy based on the low-cost biomass to construct N-containing porous composite anodes with a fast Li^+^ diffusion rate, high electronic conductivity, and outstanding structure stability.

## 1. Introduction

LIBs have been widely applied in electronic devices and other applications due to their low self-discharge rate and high power/energy density [1,2,3,4]. The anode is an important component of the LIB and has a significant influence on the cost, energy, and power density of the battery [5], but the commercial graphite anode faces great challenges in meeting the needs of high-performance LIBs owing to its low theoretical specific capacity (372 mAh g^−1^) and poor high-rate performance [6,7]. Therefore, it is essential to explore other kinds of anode materials with high electrochemical performance.

Transition metal oxide has aroused much attention to anodes for LIBs for their superior theoretical specific capacity, economic benefits, and abundant resources [8,9,10,11]. Manganese oxide is a typical transition metal oxide (TMO), which aroused much interest due to its high specific capacity and low price [12]. However, similar to other TMO-based anodes, MnO also has some serious shortcomings, including the inherent low conductivity, insufficient diffusion kinetics of Li-ions, and noticeable volume change during repeated charge and discharge, which greatly affects its future development and practical applications in energy storage.

In order to solve the above problems of MnO, many studies have focused on developing carbon-coating MnO@C composites [13]. For instance, Fu et al. used a hydrothermal technique to prepare MnO-based nanocomposites by incorporating reduced graphene oxide (rGO) into nitrogen-doped MnO and found that the lithium storage capacity of the nanocomposite reached 1020 mAh g^−1^ after 150 cycles at 0.2 A g^−1^ [14]. Qi et al. designed hierarchical MnO@C hollow nanospheres, demonstrating an excellent cycle performance, and considered that the hollow structure could lessen the volume fluctuation of MnO during lithiation [15]. Although previous studies have improved the electrochemical performance of MnO anodes to some extent, they are often obtained by complex synthesis procedures or using expensive additives such as Gr and nanotubes, which hinders their large-scale manufacturing. Consequently, it is still a major challenge to develop a simple and environmentally friendly large-scale preparation method for MnO anodes with enhanced electrochemical performance.

Recently, biomass, as the most abundant carbon material in nature, has aroused much interest [16]. It has the superiority of having low cost, abundance, being environmentally friendly, and rich in functional groups. The abundant functional groups in the biomass would offer more active sites for the carbon to coordinate with the metal ions (such as Mn ions), thus leading to a more stable structure of the composites. And the biomass always has a unique porous structure that can be replicated after calcination. The unique porous structure provides the electrode with more area to contact the electrolyte and mitigates volume expansion during charging/discharging. This enables fast electron/ion transfer and improves the cycling stability of the electrode material. It should also be mentioned that biomass-derived carbon usually has heteroatoms such as N atoms, which can both serve as the source of C and N [17]. The doping of N would improve the electron conductivity of the material by adjusting its element composition and the chemical environment of C. It can also enhance the electrolyte wettability of the carbon material, thus improving its rate performance [18].

Among the biomass materials, the tremella is one type of abundant and sustainable natural carbon-rich resources, which has outstanding water absorption and swelling rate due to the oxygen-containing groups and good porous structure. This makes the tremella a promising bio-template in the fabrication of 3D porous composites for energy storage. Considering the benefits of the tremella, this work designed a 3D porous MnO @ biomass-derived tremella carbon (MBC) containing N to overcome the shortcomings of the MnO anode. During the fabricated process, the abundant oxygen-containing groups in tremella could provide more coordination sites for Mn ions, thus promoting the strong combination of the MnO precursor between the tremella. The as-prepared MBC composite can also inherit the special porous nanostructures of the tremella, which is beneficial for the Li-ion transfer in the composites. There are abundant pyrrolic N and pyridinic N from the bio-mass tremella after high-temperature carbonation in MBC, which can improve the conductivity. The prepared MBC composites display a reversible discharge-specific capacity of about 1000 mAh g^−1^ with 98% retention after 200 cycles at 100 mA g^−1^, and the MBC electrode also exhibits excellent high-rate performance and long-cycle performance. When applied in an LFP/MBC full battery system, it delivers the discharge of 250 mAh g^−1^ at the high current density of 1000 mA g^−1^. Above all, this study proposed a simple, environmentally friendly method based on the low-cost biomass to construct N-containing MnO@C composites with fast Li^+^ transportation, high electronic conductivity, and excellent structural stability, which can effectively enhance the electrochemical performance of MnO and promote its large-scale application in LIBs. In addition, the fabrication method displays universality for other anode materials.

## 2. Results and Discussion

**Figure 1:**
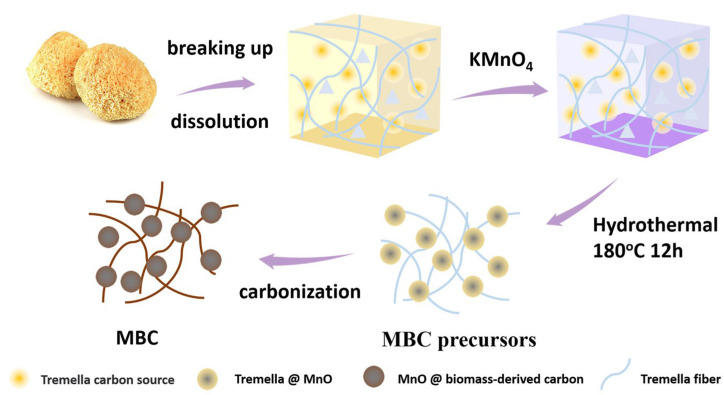
The synthesis mechanism of the MBC porous carbon.

The synthesis process is displayed in Figure 1. The MBC composite materials were fabricated through a facile one-step method. The KMnO_4_ was chosen to activate the tremella biomass to explore more active sites. At the same time, the KMnO_4_ acts as the precursor of MnO. With the effect of chemical activation of KMnO_4_ assisted with the high-temperature carbonation, MnO nanoparticles were in situ loaded on the surface of tremella-based carbon fibers. The possible activation and formation mechanism of MnO could be as follows:

(1) $$C+H_{2}O\to \;H_{2}+C$$

(2) $$2KMnO_{4}\to K_{2}MnO_{4}+MnO_{2}+O_{2}$$

(3) $$O_{2}+2C\;\to \;2CO\;$$

(4) $$MnO_{2}+C\;\to \;MnO+CO\;$$

(5) $$MnO_{2}+CO\;\to \;MnO+CO_{2}$$

(6) $$MnO_{2}+H_{2}\to \;MnO+H_{2}O\;$$

**Figure 2:**
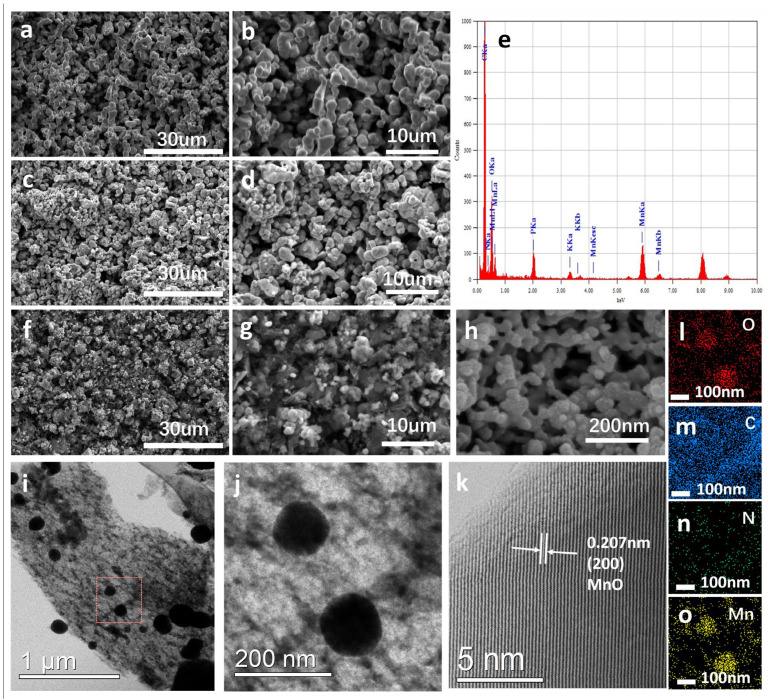
(**a**,**b**) SEM images of pure MnO; (**c**,**d**) SEM images of MSC; (**f**–**h**) SEM images of MBC with different magnifications; (**e**) EDS spectrum of the MBC; (**l**–**o**) elemental mapping images in MBC composites; (**i,k**) TEM images of MBC; (**j**) the magnification area of the red box in (**i**); (**k**) the lattice fringe of MBC with higher magnification.

The micro-morphology of MnO, MSC, and MBC are studied, as shown in Figure 2. It can be seen that the pure MnO and MSC particles are partially aggregated with larger sizes, as displayed in Figure 2a–d. In contrast, the synthesized MBC exhibits smaller and uniform nanoparticles (Figure 2f,g). The higher-magnification image shown in Figure 2h clearly exhibits the good porous structure of the MBC and the small particle with the nano size, which is beneficial for the penetration of the electrolyte and shortening the transfer pathways for Li^+^. The EDS spectrum (Figure 2e) and the elements mapping demonstrate that the C, N, O, and Mn elements are uniformly distributed in the MBC composites (Figure 2l–o). It was noted that the N element originated from the tremella biomass, which was beneficial for the improvement in electron conductivity and electrolyte affinity of the carbon materials. The TEM images shown in Figure 2i,j (the magnification area of the red box in Figure 2i) display that the MnO nanoparticles are uniformly dispersed in the carbon matrix with a diameter of about 100–200 nm. The fine structure of MnO in the MBC composites could be observed with the high-resolution TEM (Figure 2k). The clear lattice fringes with a d-spacing of 0.207 nm are visible, corresponding to the (2 0 0) plane of MnO.

**Figure 3:**
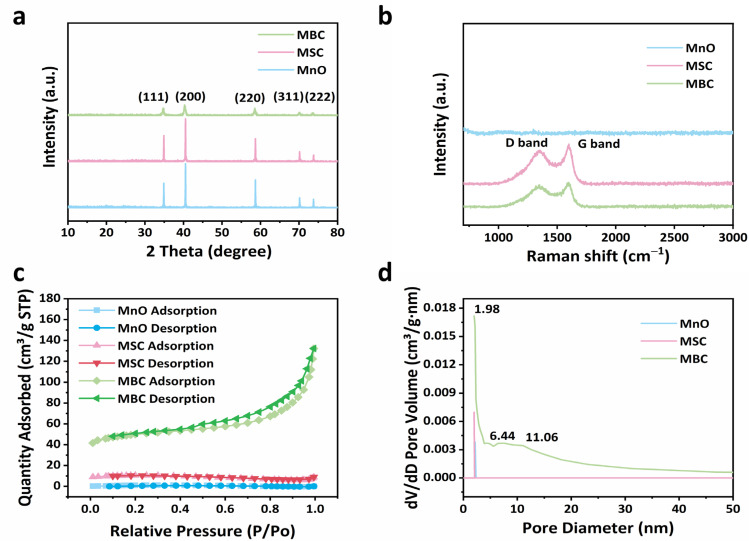
(**a**) XRD of MnO, MSC, and MBC; (**b**) Raman spectra of MnO, MSC, and MBC; (**c**) N_2_ ad-sorption/desorption curves of MnO, MSC, and MBC; (**d**) distribution of pore size in MnO, MSC, and MBC.

The crystal structure of the MnO, MSC, and MBC are shown in Figure 3a. It could be seen that all the samples show peaks of (111), (200), (220), and (311) of MnO crystals, located at about 34.7°, 40.3°, 58.3°, and 69.7°, respectively [19]. There are no obvious diffraction peaks for carbon in MSC and MBC composites, suggesting the amorphous state of the carbon. Raman spectra shown in Figure 3b display that the MSC and MBC composites both have D bands and G bands located at 1342 and 1591 cm^−1^, respectively. Typically, the ratio of the D and G bands (I_D_/I_G_) implies the order degree of the carbon materials [20]. After calculations, the I_D_/I_G_ of MBC (2.914) is a little larger than those of MSC (2.805), indicating more defected sites in MBC due to the existence of N atoms derived from the tremella. It should be mentioned that pure MnO shows no peaks of carbon in the Raman spectrum because there is no carbon in the pure MnO.

N_2_ adsorption–desorption isotherms were used to measure the porosity properties. Both curves of MnO and MSC showed the isotherm of type I, indicating the existence of little micropores (Figure 3c), but the MBC displayed type IV isotherms, suggesting the existence of mesopores in the MBC [21]. The specific surface area of MBC (155.7273 m^2^ g^−1^) is much larger than those of MnO and MSC (3.9758 m^2^ g^−1^ and 31.1993 m^2^ g^−1^), and the pore sizes distribution displayed in Figure 3d implies that there are abundant mesopores in MBC composites with the diameter of 2.0 nm, 6.4 nm, and 11.1 nm. But the pure MnO and MSC only display the distribution of little micropores. The abundant mesopores and large specific surface areas of MBC are helpful for electrolyte penetration and provide more electrochemical reaction sites.

**Figure 4:**
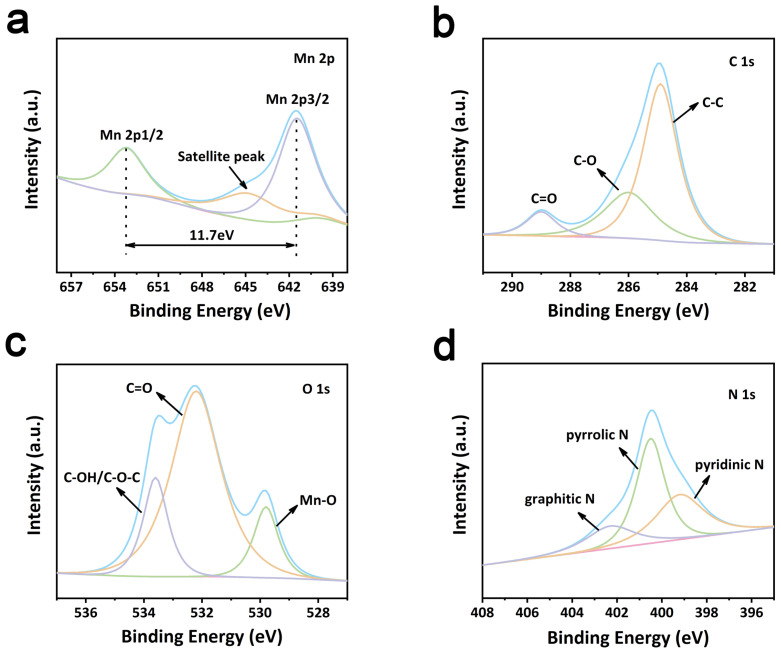
(**a**) Mn 2p spectra of MBC (the blue line is the overall spectrum of Mn 2p; the green line is the Mn 2p1/2; the purple line is the Mn 2p3/2; the orange line is the Satellite peak); (**b**) C 1s spectra of MBC (the blue line is the overall spectrum of C 1s; the green line is the C-O; the purple line is the C=O; the orange line is the C-C); (**c**) O 1s spectra of MBC (the blue line is the overall spectrum of O 1s; the green line is the Mn-O; the purple line is the C-O-C and C-OH; the orange line is the C=O); (**d**) N 1s spectra of MBC (the blue line is the overall spectrum of N 1s; the green line is the pyrrolic N; the purple line is graphitic N; the orange line is the pyridinic N).

Figure 4a displays the XPS spectra of Mn 2p with high resolution in MBC composites. There are two obvious peaks at ~652.4 eV and ~642.3 eV, which are ascribed to Mn (2p1/2) and Mn (2p3/2) [22]. The high-resolution C 1s spectrum for the MBC sample is associated with the binding energy of C=O (287.3 eV), C-C (283.5 eV), and C-O (285.1 eV), respectively (Figure 4b). The O 1s spectrum displays the C-O, Mn-O, and O-C=O bonds at 531.4 eV, 530.0 eV, and 532.8 eV (Figure 4c), respectively, implying that O atoms are bonded with C and Mn atoms. In addition, three peaks of graphite N, pyrroline N, and pyridine N were observed in the fitted XPS spectra of N 1s at 402.5 eV, 400.6 eV, and 398.1 eV, respectively (Figure 4d) [23]. In Table 1, pyridine-N and pyrrole-N have a large proportion of the total concentration. Pyrrole-N and pyridine-N can help to improve the electronic conductivity of the materials [24].

**Figure 5:**
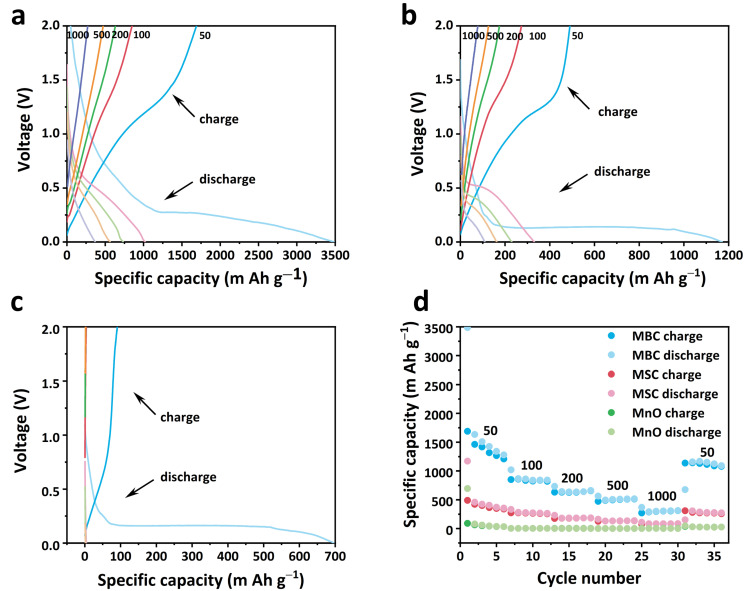
Charge-discharge curves of (**a**) MBC (the blue lines are the charge-discharge curves at 100 mA g^−1^; the red lines are the charge-discharge curves at 200 mA g^−1^; the green lines are the charge-discharge curves at 500 mA g^−1^; the orange lines are the charge-discharge curves at 1000 mA g^−1^; the purple lines are the charge-discharge curves at 2000 mA g^−1^); (**b**) MSC (the blue lines are the charge-discharge curves at 100 mA g^−1^; the red lines are the charge-discharge curves at 200 mA g^−1^; the green lines are the charge-discharge curves at 500 mA g^−1^; the orange lines are the charge-discharge curves at 1000 mA g^−1^; the purple lines are the charge-discharge curves at 2000 mA g^−1^); (**c**) MnO from 100 to 1000 mA g^−1^ (the blue lines are the charge-discharge curves at 100 mA g^−1^; the red lines are the charge-discharge curves at 200 mA g^−1^; the green lines are the charge-discharge curves at 500 mA g^−1^; the orange lines are the charge-discharge curves at 1000 mA g^−1^; the purple lines are the charge-discharge curves at 2000 mA g^−1^); (**d**) Rate performance of MnO, MSC, and MBC from 100 to 1000 mA g^−1^.

To investigate the electrochemical performance of MnO, MSC, and MBC, coin cells (CR2016) were assembled with MnO, MSC, or MBC as the anodes. Figure 5a displays the charge/discharge curves of MBC under different current densities [25]. It can be observed that the initial discharge plateau of MBC at 100 mA g^−1^ displays a platform around 0.24 V, which may be related to the reduction from Mn^2+^ to Mn^0^ accompanied by the Li_2_O formation, and this peak shifts to 0.5 V in the next cycles, suggesting the irreversible structural change due to the formation of metallic manganese and Li_2_O. And then, the initial charging plateau is at ~1.26 V, which could be attributed to the oxidation of Mn^0^ to Mn^2+^ again [26]. The charge–discharge curve of the MSC is similar to that of the MBC (Figure 5b), but the voltage plateaus of pure MnO almost disappear under the following high current density (Figure 5c), which suggests the high polarity of the pure MnO under high rates. The detailed rate capability of the three samples is displayed in Figure 5d. The discharge capacity of MBC at 100 mA g^−1^ reaches 3500 mAh g^−1^, and the reversible capacity can also reach up to 1200 mAh g^−1^, which is much higher than those of the MSB and MnO (500 and 50 mAh g^−1^). And it should be mentioned that the lost irreversible capacity could be divided into two parts: the side reaction of the irreversible process involving electrolyte decomposition; and the formation of SEI layers [27]. The reversible discharge capacity of MBC was 800, 500, 300, and 120 m Ah g^−1^ at 200, 500, 1000, and 2000 mA g^−1^, respectively, and the specific capacity can recover to 1200 mAh g^−1^ when the current density returns to 100 mA g^−1^, suggesting the good reversibility of MBC electrode. However, the capacity of pure MnO reduced to almost 0 mAh g^−1^ when the current density was higher than 200 mA g^−1^, indicating the poor rate property. The good rate property of MBC anode may be attributed to three reasons: on the one hand, the rich mesopores and high porosity are beneficial for the Li^+^ transport in the composites; on the other hand, the tremella-derived carbon with N doping improves the electrons conductivity of the materials, enabling the fast electrons transportation; thirdly, the doping of N atoms may enhance the electrolyte affinity of carbon material so as to improve its rate performance.

**Figure 6:**
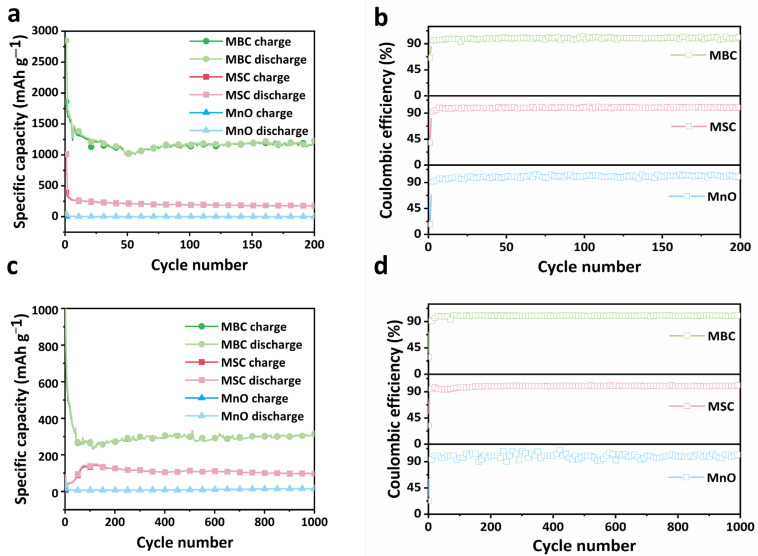
Cycling performances of MnO, MSC, and MBC at (**a**) 100 mA g^−1^ and (**c**) 1000 mA g^−1^. Coulombic efficiency of MnO, MSC, and MBC at (**b**) 100 mA g^−1^ and (**d**) 1000 mA g^−1^.

Cycle performance is important for anodes, as displayed in Figure 6. As shown in Figure 6a, the discharge-specific capacity of the MBC remains at 1200 mAh g^−1^ with an initial coulomb efficiency (CE) of 69% (Figure 6b). The low initial CE is a common phenomenon of various conversion-type electrodes, where the irreversible insertion defects of lithium within the carbon, the establishment of the SEI layer, and electrolyte loss are the three main factors that lead to an irreversible capacity drop [28]. The MBC keeps a 99% capacity retention after 200 cycles at 100 mA g^−1^ with coulombic efficiency close to 100% (Figure 6b), indicating the excellent stability of the MBC electrode. As for the MSC anode, it only retains 55% capacity after 200 cycles. As for the MnO anode, the discharge capacity nears 0 m Ah g^−1^ after 200 cycles. We further investigated the long-cycle performance of the anodes at a high current of 1000 mA g^−1^. As shown in Figure 6c, the capacity of the MBC anode is still 300 mAh g^−1^ with 99% capacity retention (relative to the capacity second cycle) after long 1000 cycles, which indicates outstanding cycling stability and shows good application value of the MBC electrode. MBC anodes have good cycling stability because their porous structure reduces the volume expansion during cycling. More importantly, in the literature listed in Table 2 [29,30,31,32,33,34,35,36,37,38], the electrochemical performance of MBC anodes is better than some anodes reported in other sources of the literature.

**Figure 7:**
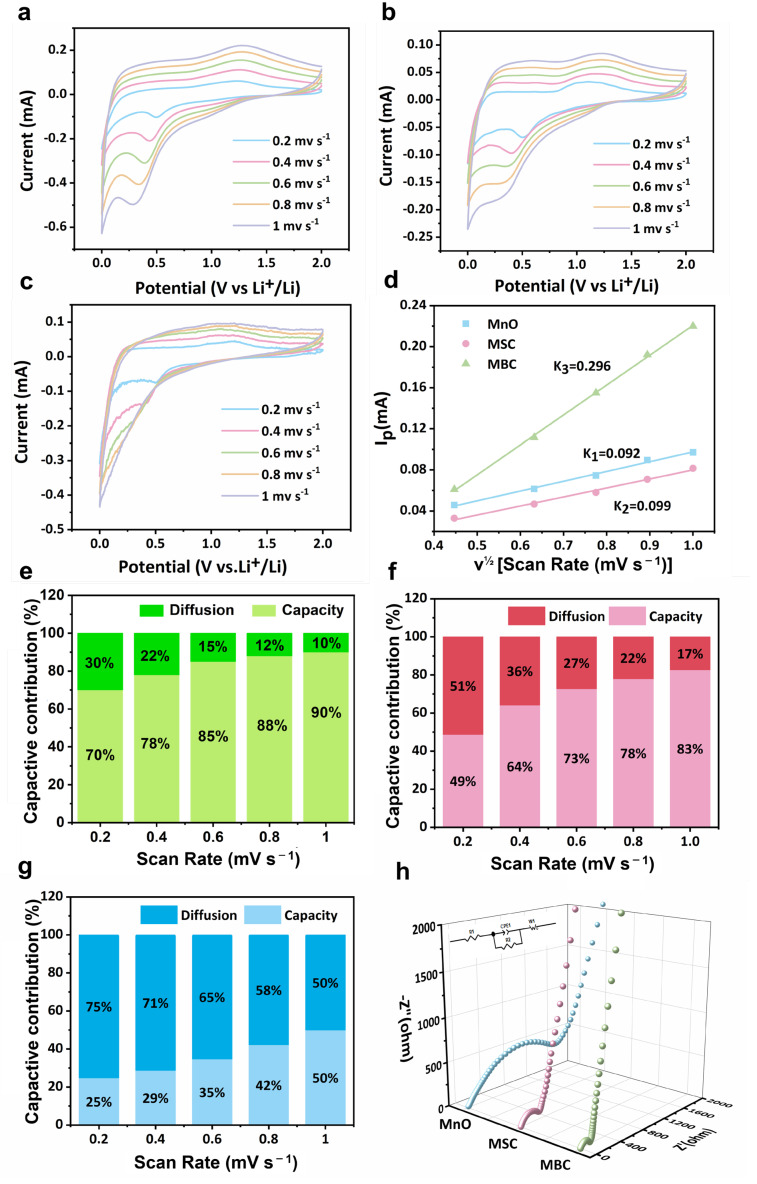
CV curves of (**a**) MBC, (**b**) MSC, and (**c**) MnO from 0.2 to 1 mV s^−1^; (**d**) the fitting line of the relationship between the Ip and v^1/2^; (**e**) capacitive contribution of MBC; (**f**) capacitive contribution of MSC; (**g**) capacitive contribution of MnO; (**h**) EIS of the samples (inset is the equivalent circuit).

We further analyzed the kinetic processes of the cell to analyze the good performance of the MBC anode. Figure 7a–c displays the CV curves under different current densities. As shown in Figure 7a, there are two clear reduction peaks at about 0.06 V and 0.36 V for MBC, which are the formation of Li_2_O and the reduction in MnO to manganese. During the anodic scan, there is a visible oxidation peak centered at approximately 1.25 V, which corresponds to the oxidation process occurring when Mn is being converted into MnO [39]. Similar peaks are observed for MSC and MnO, indicating a similar reaction mechanism. In addition, the peak of the current density in CV curves was shown as follows [2]:

*Ip =* 2.6 *×* 10^5^*n*^3/2^*D*^1/2^*AC_o_v*^1/2^ (7)

The fitting lines based on the Ip and the v^1/2^ are displayed in Figure 7d. The slopes of the fitting lines for MBC, MSC, and MnO (K3, K2, and K1) were 0.296, 0.099, and 0.092. After calculation, the diffusion coefficient of Li^+^ (D) in the MBC was 8.38 × 10^−11^ cm^2^ s^−1^, which was much higher than that of the MSC and MnO (9.38 × 10^−12^ cm^2^ s^−1^ and 8.09 × 10^−12^ cm^2^ s^−1^). The higher Li^+^ transportation rate in the MBC electrode was because the abundant mesopores were beneficial for electrolyte penetration, and the N-doping could also enhance the electrolyte affinity of the composites.

The Li^+^ storage mechanism has two main behaviors, including intercalation behavior and capacitive effect. It is shown as follows:

(8) $$i\left(v\right)=av^{b}$$

(9) $$Log\left(i\right)=Log\left(a\right)+bLog(v)$$

Figure 7e–g shows the capacitive contribution in the three anodes after calculation. It can be observed that the MBC has a much higher capacitive contribution than the MSC and MnO, and with the scanning rate increasing, the capacitive contribution in MBC can reach 90%, which is beneficial for the enhancement of the high-rate performance of the anode [40].

EIS was performed to investigate the resistance of charge transfer (*R_ct_*) and the diffusion rate of Li^+^ in the electrode. The MBC in Figure 7f displays an *R_ct_* of 150 Ω, which is small compared to the *R_ct_* of MSC and MnO (300 Ω and 1000 Ω), suggesting that the tremella-based carbon framework could improve the electronic conductivity of the composites and reduce the *R_ct_* at the electrolyte/electrode interface. Above all, the kinetic analysis results indicated that the MBC had fast electron and Li^+^ transport rates, thus contributing to the outstanding performance.

**Figure 8:**
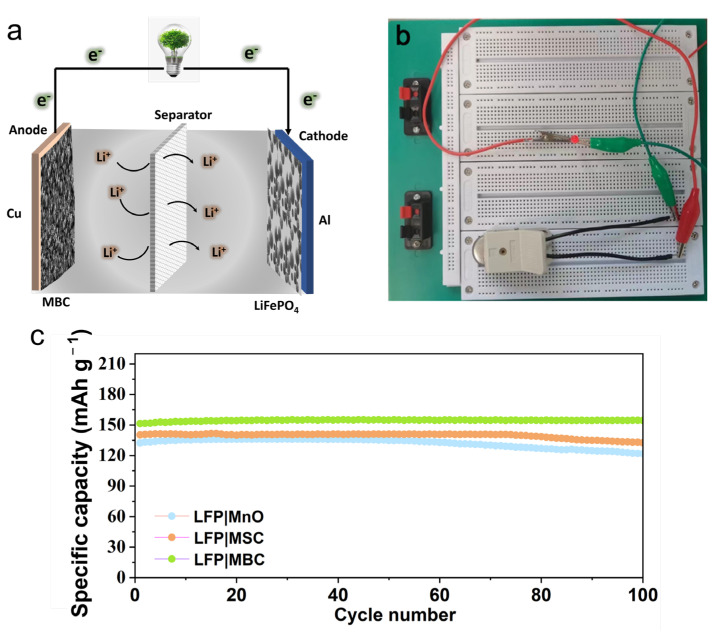
(**a**) schematic of the full battery; (**b**) coin full cell light up the picture of LED lights (**c**) cycle performance of the full battery at 100 mA g^−1^.

Considering the practical application, we fabricated coin full cells with LiFePO_4_ as the cathode and the MBC as the anode, as shown in Figure 8a. The full-cell with MBC anode presents a much higher capacity of 250 mAh g^−1^ than the batteries with MSC and pure MnO as anode. After 200 cycles, a full battery with an MBC anode still maintains 205 mAh g^−1^ with 85% capacity retention (Figure 8c), revealing good cycle stability of the LiFePO_4_/MBC full cell. Furthermore, we lightened a white LED bulb with the LiFePO_4_/MBC full cell (Figure 8b) after being fully charged, which suggested the promising application prospect of MBC as the anode for LIBs.

## 3. Materials and Methods

### 3.1. Sample Synthesis

Firstly, break up the dried tremella and pass it through 200 mesh sieves. Then, immerse 3 g of screening tremella powder into 40 mL of deionized water with stirring to form a homogeneous hydrogel. Next, 3 g KMnO_4_ was put into the tremella hydrogel, and the homogeneous KMnO_4_/tremella hydrogel was obtained. Then, the mixtures were put into the Teflon container at 180 °C for 12 h. After cooling, the MnO @ biomass-derived carbon precursors were washed three times with distilled water and alcohol by filtration, followed by drying at 60 °C. Finally, the MBC composites were obtained by calcination at 800 °C under N_2_ atmosphere.

As a comparison, the pure MnO and MnO@sucrose composites (MSC) with sucrose as the common carbon source were produced with the same synthesis process without adding tremella. It should be mentioned that sucrose was chosen as a common carbon source for comparation.

### 3.2. Physical Characterization

Surface morphologies were investigated by scanning electron microscopy (SEM, JSM-7800F, Tokyo, Japan); microstructures were characterized by transmission electron microscopy (TEM, JEM-2100(UHR), Philips, Japan), and the pore size distribution was implemented using BET analyzer (micromeritics, ASAP, 2020, Norcross, GA, USA). Raman spectrums were recorded on a Bruker Optics Spectrometer. The X-ray diffraction (XRD, XRD-6100, Tokyo, Japan) patterns were conducted with the 2θ range of 10–80°. In addition, the surface electronic state was surveyed with X-ray photoelectron spectroscopy (XPS, PHI500, Beijing, China).

### 3.3. Electrochemical Characterization

The electrochemical properties were measured with the CR2016 coin cell using lithium metal as another electrode. The coin cells were assembled in the N_2_-filled glove box (Mikrouna, Shanghai, China). A mixture with active materials, Super P and polyvinylidene fluoride (PVDF) adhesive (mass ratio was 8:1:1), was pasted onto the copper foil and then dried at 90 °C for 10 h. Afterward, the anode slices were cut into round disks with a diameter of 12 mm. The mass loading of the active material was about 1.0–1.5 mg cm^−2^. The charge and discharge measurements were conducted on the LAND test system (CT2001A, LAND, Wuhan, China). The cyclic voltammetry (CV) curves and electrochemical impedance spectroscopy (EIS) were tested on an electrochemical workstation (CHI604E) with a frequency range of 10^5^–10^−1^ Hz.

## 4. Conclusions

In conclusion, the MnO@C composites anode from the tremella (MBC) was successfully designed by a low-cost one-pot method. Chemical activation with KMnO_4_ was employed to convert the tremella into MnO @ biomass-derived tremella carbon (MBC). At the same time, the KMnO_4_ can be converted into MnO active material. As a result, MnO nanoparticles were loaded on the surface of the tremella-derived carbon matrix uniformly. The obtained MBC showed abundant distribution of mesopores with a high surface area of 155.7273 m² g^−1^. As the anode for LIBs, the MBC at 100 mA g^−1^ displayed a high specific capacity of 1200 m Ah g^−1^ with 99% capacity retention after 200 cycles, which is better than many other anodes previously reported in the literature. When used in the LFP/MBC full battery system, it delivers a high reversible specific capacity of 250 mAh g^−1^ at 1000 mA g^−1^. The anode material was shown to have good potential for application in advanced LIBs by electrochemical performance. And this study offers a low-cost and effective strategy to fabricate high-performance anodes for LIBs.

## Figures and Tables

**Table 1:** The content of the N 1s peaks in samples.

B.E. (eV)	N1 (402.3)	N2 (400.5)	N3 (399.2)
Assignment	graphitic N	pyrrolic N	pyridinic N
MBC	17.2	48.3	34.5

**Table 2:** The comparison of the MBC with the other MnO composites reported in other sources of the literature.

Samples	Current Density (mA g^−1^)	Specific Capacity (mAh g^−1^)	Cycling Performance	References
Porous MnO/C microspheres	100	812	98% 100 cycles	[29]
Hollow porous MnO/C	100	740	99% 50 cycles	[30]
3D porous MnO/C	100	846	99% 100 cycles	[31]
MnO/C porous microspheres	100	525	93% 100 cycles	[32]
MWNTs/MnO rods/C	210	658	99% 200 cycles	[33]
Porous MnO microsphere	50	700	88% 50 cycles	[34]
Microsized porous MnO/C	100	818	99% 100 cycles	[35]
CF@MnO	100	734	99% 200 cycles	[36]
MnO/rGO	100	846	99% 110cycles	[37]
NLIG	200	699	70% 400 cycles	[38]
MBC	100	1200	99% 200 cycles	This work

## Data Availability

The authors confirm that the data supporting the findings of this study are available within the article.
